# Humanizing pediatric anesthesia care: development and validation of an educational video for caregivers^✰^^✰✰^

**DOI:** 10.1016/j.jped.2026.101602

**Published:** 2026-09-08

**Authors:** Juliana Lais Carneiro, José Eduardo Ferreira Manso, Ivonete Siviero

**Affiliations:** a Postgraduate Program in Surgical Sciences, Department of Surgery, Faculty of Medicine, Federal University of Rio de Janeiro (UFRJ), Rio de Janeiro, RJ, Brazil; b Department of Surgery, Faculty of Medicine, Federal University of Rio de Janeiro (UFRJ), Rio de Janeiro, RJ, Brazil; c Division of Pediatric Surgery, Department of Surgery, Faculty of Medicine, Federal University of Rio de Janeiro (UFRJ), Rio de Janeiro, RJ, Brazil; d Anesthesiologist, Instituto de Puericultura e Pediatria Martagão Gesteira (IPPMG), Federal University of Rio de Janeiro (UFRJ), Rio de Janeiro, RJ, Brazil

**Keywords:** Pediatric anesthesia, Educational technology, Patient-centered care, Caregivers

## Abstract

**Objective:**

To develop an educational video on pediatric anesthesia, obtain evidence of content validity from experts, and assess its clarity, adequacy, and acceptability among caregivers.

**Methods:**

This methodological study was conducted at a tertiary pediatric university hospital in Brazil between 2023 and 2025 and comprised sequential qualitative and quantitative phases. The qualitative and development phases included semistructured interviews with 10 caregivers, a narrative literature review, script development, and production of a preliminary video. The validation phases included 22 experts and 12 caregivers, who completed different instruments according to the objectives of each assessment. Internal consistency was assessed using Cronbach’s alpha, and content validity was assessed using the Scale-level Content Validity Index using the average method (S-CVI/Ave).

**Results:**

Caregiver interviews identified information needs related to fasting, type of anesthesia, monitoring, and postoperative care. Using the expert instrument, the S-CVI/Ave was 94%, and Cronbach’s alpha was 0.94. Using the caregiver instrument, the corresponding values were 98% and 0.75. These findings should be interpreted separately because different instruments were used. Suggestions from both groups were subsequently incorporated into the final video, which was produced in Brazilian Portuguese, lasted 4 min and 40 s, and used 2D animation, illustrations, text, and narration.

**Conclusions:**

The evaluated version achieved satisfactory content validity indices and was considered clear, relevant, and appropriate for caregivers. The revised final version was not reassessed. Clinical effectiveness was not evaluated and should be investigated in future studies.

## Introduction

Pediatric surgical procedures are common, and preoperative anxiety among children and their caregivers may adversely affect clinical outcomes [1]. Clear perioperative information may reduce anxiety [2,3], which has been associated with lower analgesic use, shorter hospital stays, and fewer postoperative maladaptive behaviors [4]. Videos may improve comprehension and have been used internationally to prepare children and caregivers for pediatric anesthesia [5,6]. However, the literature search did not identify another formally validated educational video specifically addressing pediatric anesthesia for caregivers. Therefore, this study aimed to develop an educational video, assess its content validity with experts, and evaluate its suitability among caregivers.

## Methods

This multiphase methodological study, conducted between April 2023 and December 2025, comprised a qualitative development phase followed by quantitative validation with experts and assessment by the target audience. It included six steps: 1) semistructured interviews with caregivers, 2) a narrative literature review, 3) development of the script and preliminary video, 4) expert validation, 5) target-audience validation and 6) video finalization after the suggested adjustments. The study aimed to develop an educational video, obtain evidence of its content validity through expert evaluation, and assess its suitability among caregivers. The study was approved by the Research Ethics Committee of the Instituto de Puericultura e Pediatria Martagão Gesteira, Universidade Federal do Rio de Janeiro (IPPMG/UFRJ) (Approval No 5.952.452), and written informed consent was obtained from all participants in Steps 1, 4, and 5.

Reporting of the educational intervention development was guided by the *Guideline for Reporting Evidence-based Practice Educational Interventions and Teaching* (GREET) [7], whose checklist is provided in Supplementary Table S1.

### Step 1: Semistructured interviews with caregivers

The interviews, conducted between April and July 2023, identified information gaps and needs that informed the educational video. Reporting of the qualitative component was guided by the *Consolidated Criteria for Reporting Qualitative Research* (COREQ) [8], whose checklist is provided in Supplementary Table S2.

Caregivers aged 18 years or older who accompanied children scheduled for elective surgery were recruited by convenience sampling during the preanesthetic assessment in the pediatric inpatient unit of a Brazilian tertiary university hospital.

Caregivers were excluded if the child had a chronic condition or a history of multiple surgeries, or if literacy, communication, or cognitive limitations prevented them from understanding the consent form, providing informed consent, or responding coherently to the interview. These criteria were assessed pragmatically, without formal health literacy or cognitive instruments.

Data were collected using a semistructured interview guide developed by the authors based on the literature and the clinical experience of the research team. The instrument included sociodemographic questions and four open-ended questions about the preanesthetic period, the anesthetic procedure, the postoperative period, and the educational video, as well as 16 probing questions used when necessary. The guide was pretested with two caregivers, resulting in minor language adjustments, and their data were not included in the final analysis. The complete interview guide is available in Supplementary Appendix 1.

The interviews were conducted by the principal investigator, an anesthesiologist experienced in pediatric anesthesia and familiar with the clinical setting, who had no prior relationship with the participants and no involvement in their clinical care or decision-making. After consent was obtained, the interviews were audio-recorded, transcribed, and anonymized. Thematic saturation was monitored through field notes, concurrent reviews, preliminary coding, and recurrence of responses. It was reached at the eighth interview and confirmed in the ninth and tenth, which yielded no new concerns, informational needs, codes, or categories. Data collection was therefore concluded after ten interviews, consistent with saturation criteria described in the qualitative research literature [9,10]. Under the supervision of a senior researcher, the principal investigator conducted thematic content analysis following Bardin’s framework. The analysis included transcript reading, identification of meaning units, coding, grouping into thematic categories, and interpretation of the data. ATLAS.ti®, version 25, was used solely to organize, code, and retrieve the data.

Categories were descriptively quantified as counts and percentages of the total units of meaning to identify the most frequent information needs and inform the video content [11,12]. Percentages were calculated based on the total number of coded meaning units, without inferential purposes.

### Step 2: narrative literature review

The narrative review was conducted between August and December 2023 and updated in 2025 to support the scientific and educational content of the video. Searches were performed in PubMed, SciELO, and LILACS using combinations of the following terms in Portuguese, English, and Spanish: “pediatric anesthesia,” “health education,” “educational video,” “preoperative preparation,” “preoperative education,” “caregivers,” “parents,” and “parental anxiety”.

Recent publications were prioritized, along with relevant guidelines and seminal studies. The selection addressed caregivers’ information needs, communication, preoperative preparation, anxiety reduction, and pediatric anesthesia safety. The findings, together with the qualitative data obtained from caregivers, informed the video script and storyboard.

### Step 3: development of the preliminary video

The script and preliminary video were developed between January and December 2024. The script followed a four-column audiovisual format created for this project, comprising the 1) scene number, indicating its sequence; 2) on-screen text, containing all written information displayed; 3) visual description, detailing the setting, characters, actions, animations; and 4) narration, containing the exact voice-over text. It also specified the characters, setting, visual identity, animations, narration style and other technical elements to ensure consistency across the script, storyboard, and final video. The video was produced in Brazilian Portuguese by a company specializing in professional audiovisual content.

### Step 4: expert validation

The initial version of the video was evaluated by experts between January and June 2025 using a structured instrument in a single round, during which all participants assessed the same version.

The experts were selected using an adapted Fehring scoring system [13], considering academic qualifications, professional experience, and scientific production in the field. Potential experts were identified through the Lattes Platform and peer recommendations.

After accepting the invitation by email, the experts received the preliminary version of the video and the instrument validated by Teixeira and Mota [14], which consisted of 21 items distributed across three domains: objectives, structure and presentation, and relevance. The instrument assessed the content validity of the educational material using a four-point Likert scale: Totally Adequate (TA), Adequate (A), Partially Adequate (PA), and Inadequate (I), with additional space for suggestions.

Internal consistency was assessed using Cronbach’s alpha and interpreted descriptively as exploratory. I-CVI was the proportion of “Totally Adequate” or “Adequate” ratings; domain CVIs were mean I-CVIs, and S-CVI/Ave and S-CVI/UA summarized scale-level validity. I-CVI ≥ 0.78 and S-CVI/Ave ≥ 0.90 were considered satisfactory [15]. Modified kappa adjusted for chance agreement and was interpreted as fair (0.40–0.59), good (0.60–0.74), or excellent (> 0.74) [16]. Ninety-five percent confidence intervals were calculated for item-, domain-, and scale-level estimates. Analyses were performed using R version 4.5.1.

### Step 5: target audience validation

Validation with family members of patients hospitalized for surgical treatment was conducted between July and August 2025, using the same eligibility criteria as in Step 1. After informed consent was obtained, the video was individually presented on an electronic device in a single session, followed by administration of the Teixeira and Mota instrument [14], which consisted of 25 items distributed across the domains of objectives, organization, style, appearance, and motivation. The instrument assessed the semantic and face adequacy of the video for the target audience using the previously described Likert scale and included a space for suggestions.

For target audience validation, the same statistical procedures used for the expert panel were applied to assess the semantic and face adequacy of the video. These included Cronbach’s alpha for internal consistency, the I-CVI for each item, mean I-CVIs for each domain, the S-CVI/Ave and S-CVI/UA for overall adequacy, 95% confidence intervals for item-, domain-, and scale-level indices, and the modified kappa coefficient (κ*) for each item to account for chance agreement.

### Step 6: video finalization

The final version of the educational video was produced between September and December 2025, based on responses to the validation questionnaires, the CVI calculations, and suggestions from the experts and target audience.

## Results

### Step 1: semistructured interviews with caregivers

Ten family members participated in the study, with a median age of 34.0 years (IQR: 27.8–39.0); 40.0% were aged 25–30 years, 40.0% were aged 31–40 years, and 20.0% were older than 40 years. The median patient age was 5.5 years (IQR: 4.0–7.8); 40.0% were aged 0–4 years, 30.0% were aged 5–7 years, and 30.0% were aged 8–9 years. Regarding their relationship with the patient, 70.0% of participants were mothers and 30.0% were fathers. Regarding educational attainment, 10.0% had completed elementary school, 10.0% had incomplete high school education, 60.0% had completed high school, and 20.0% had completed higher education.

The content analysis identified recurring questions and information needs regarding the perioperative anesthetic process. Descriptive frequencies indicated the recurrence of the categories. Preoperative fasting was the most frequent topic, accounting for 16 meaning units (28.6%) related to its duration, rationale, and whether water or other liquids could be consumed. Intraoperative monitoring and type of anesthesia each accounted for nine meaning units (16.1%); postoperative agitation accounted for eight (14.3%); shivering or feeling cold accounted for five (8.9%); and postoperative pain, allergy to anesthesia, and intraoperative awareness each accounted for three (5.4%). These categories guided the selection and organization of the video content. Representative excerpts and their contributions are presented in Supplementary Table S3.

### Step 2: narrative literature review

The narrative review identified complementary evidence regarding caregivers’ information needs and the technical and scientific foundations of pediatric anesthesia. The main findings are summarized in Supplementary Table S4.

The studies showed that family members seek clear, timely, and accessible information about fasting, anesthesia, the operating room, procedure duration, risks, adverse effects, pain, recovery, and postoperative care [2,17,18]. They also identified knowledge gaps [19] and highlighted the use of educational materials, such as videos and printed brochures, as a vital complement to verbal guidance, in order to improve comprehension, ensure safety, and increase overall family satisfaction [6,18,19].

Textbooks, guidelines, consensus statements, and reviews addressing preoperative assessment, fasting, respiratory symptoms, general, inhalational, and regional anesthesia, analgesia, nausea and vomiting, emergence delirium, and postanesthesia recovery ensured technical accuracy, alignment with current recommendations, and responsiveness to caregivers’ needs [[20], [21], [22], [23]].

### Step 3: development of the preliminary video

The video script was organized into four columns and 20 scenes based on family members’ questions, the prior knowledge of the authors and institutional professionals, and the literature review. The first version was a 3-minute and 40-second 2D animation integrating illustrations, text, and narration to facilitate comprehension.

### Step 4: expert validation

Of the 37 invited experts, 22 completed the video assessment in a single round, yielding a 59.5% response rate. Of these, 15 scored above five on the adapted Fehring scale, while seven with lower scores were retained because of relevant clinical or academic expertise. Panel characteristics are presented in Supplementary Table S5.

The questionnaire showed high internal consistency (Cronbach’s alpha = 0.94). I-CVI values ranged from 77.3% to 100%. Mean domain-level CVIs were 97.3% (95% CI: 92.2–99.4) for objectives, 92.1% (95% CI: 88.0–95.2) for structure and presentation, and 94.5% (95% CI: 88.5–98.0) for relevance. The S-CVI/Ave was 94% (95% CI: 91.4–95.9), and the S-CVI/UA was 23.8%. Modified kappa values ranged from 0.771 to 1.000, indicating agreement beyond chance.

Item 2.4, on sociocultural appropriateness, had the lowest score, with 17 of 22 favorable ratings (I-CVI = 77.3%; 95% CI: 54.6–92.2; κ* = 0.771). Because it was below the predefined cutoff, the video language was revised to improve clarity, accessibility, and sociocultural appropriateness. Item-level results are shown in Table 1, and the suggested modifications in Table 2.

**Table 1:** Content validity indices and modified kappa coefficients for the expert panel.

Item	CVI (%)	95% CI (%)	Modified kappa, κ*
**OBJECTIVES**	**97.3**	**92.2–99.4**	**—**
1.1) The information/content of the video is consistent with the daily needs of families in the preoperative period.	95.5	77.2–99.9	0.955
1.2) The information/content is important for the relatives of patients in the preoperative period.	100.0	84.6–100.0	1.000
1.3) The video encourages and/or prompts changes in behavior and attitude among families in the preoperative period.	100.0	84.6–100.0	1.000
1.4) The video is suitable for circulation in the scientific community as a health education strategy.	95.5	77.2–99.9	0.955
1.5) The video meets the objectives of institutions that work with patient families in the preoperative period.	95.5	77.2–99.9	0.955
**STRUCTURE AND PRESENTATION**	**92.1**	**88.0–95.2**	**—**
2.1) The video is appropriate for families of children undergoing elective surgery in the preoperative period.	100.0	84.6–100.0	1.000
2.2) The messages are presented in a clear and objective manner.	86.4	65.1–97.1	0.864
2.3) The information presented in the video is scientifically correct.	95.5	77.2–99.9	0.955
2.4) The video is appropriate for the sociocultural level of families of children undergoing elective surgery in the preoperative period.	77.3	54.6–92.2	0.771
2.5) There is a logical sequence of the content proposed in the video.	100.0	84.6–100.0	1.000
2.6) The information contained in the video is well-structured in terms of grammar and spelling.	95.5	77.2–99.9	0.955
2.7) The writing and speaking style correspond to the families' level of knowledge.	86.4	65.1–97.1	0.864
2.8) The sizes of the title, text, and topics are adequate.	95.5	77.2–99.9	0.955
2.9) The video illustrations are expressive and sufficient.	86.4	65.1–97.1	0.864
2.10) The video is appropriate.	95.5	77.2–99.9	0.955
2.11) The video duration is adequate.	95.5	77.2–99.9	0.955
**RELEVANCE**	**94.5**	**88.5–98.0**	**—**
3.1) The themes addressed in the video depict key aspects that should be reinforced when guiding families of children in the preoperative period of elective surgeries.	95.5	77.2–99.9	0.955
3.2) The video allows for the generalization and transfer of learning to different contexts, including different health education settings.	100.0	84.6–100.0	1.000
3.3) The video promotes knowledge construction.	95.5	77.2–99.9	0.955
3.4) The video addresses the necessary topics for the understanding of families of children in the preoperative period of elective surgeries.	95.5	77.2–99.9	0.955
3.5) The video is suitable for use by any professional working with families of children in the preoperative period of elective surgeries.	86.4	65.1–97.1	0.864
**S-CVI/Ave**	**94.0**	**91.4–95.9**	**—**
**S-CVI/UA**	**23.8**	**—**	**—**

Note: I-CVI, Item-level Content Validity Index; S-CVI/Ave, Scale-level Content Validity Index using the average method; S-CVI/UA, Scale-level Content Validity Index using the universal agreement method; CI, confidence interval; κ*, modified kappa coefficient. I-CVI was based on ratings of Totally Adequate or Adequate, and domain values represent mean I-CVIs. Exact binomial 95% CIs were calculated for individual items and pooled favorable ratings for domain and scale estimates. κ* was calculated only for individual items; —, not applicable. Complete response distributions are provided in Supplementary Table S7. N = 22 experts.

**Table 2:** Summary of the qualitative analysis of changes suggested by experts and the target audience.

Suggestions from experts and the target audience	Change implemented	Justification
[…] add subtitles for the hearing impaired.	Yes	To ensure inclusivity for hearing-impaired individuals.
[…] the child's father could wear less formal clothing.	Yes	To better reflect the reality of the target audience.
[…] the toy used to distract the child could be something else (other than a screen).	Yes	To discourage the use of screens/electronic devices.
[…] I suggest the family arrives at the hospital on foot rather than by car.	Yes	To better reflect the reality of the target audience.
[…] it could be explicit that these are elective procedures.	Yes	Urgent procedures follow a different care workflow.
[…] pre-anesthetic medication could be added.	Yes	Although not routine at this institution, it is used in many hospitals and was therefore included.
[…] regarding the part "when the stomach is empty, we reduce the risk of aspiration of food and liquids, which can cause serious complications." I think this could be better explained.	No	For the target audience, a clinical explanation of bronchoaspiration mechanisms is not justified.
[…] some explanations on why fasting is important might not be clear enough for people with lower education levels. The part regarding aspiration might be misunderstood.	Yes	Narration was altered to improve comprehension by the target audience.
[…] regarding the use of preoperative medications, it is suggested to "follow the team's guidance." It would be interesting to specify which team.	Yes	To make the information clearer for the target audience.
[…] regarding anesthesia, it is explained that there are different types (general, regional), and you exemplify inhalational. To me, it was a bit confusing. Is inhalational general? A type of general?	Yes	To clarify the types of anesthesia and which one is being shown in the video.
[…] regarding the choice of anesthesia, it is stated that it is based on the patient's needs. What are those needs?	No	There is a wide range of issues that are too technical for the target audience.
[…] you specifically mention inhalational. Is it the most used in children?	Yes	To clarify which anesthesia is most commonly used in children.
[…] "a gas with a different smell," this might cause discomfort. What kind of different smell? Is it good or bad?	Yes	The phrase "different smell" and other adjectives regarding the odor were removed.
[…] is your video intended for a specific age group?	No	The video is aimed at caregivers and does not have a specific age range.
[…] I suggest you do not use the terms aspiration, block, and monitoring, as the target audience likely will not understand them.	Yes	Terms were altered to facilitate comprehension by the target audience.
[…] some language issues could be improved for laypeople, who generally do not know what vital signs, monitoring, or blocks are.	Yes	Terms were altered to facilitate comprehension by the target audience.
[…] meal images are not very clear (especially breast milk).	Yes	Images were enlarged.
[…] identification wristband.	Yes	Although not routine in all hospitals, it was added to highlight the importance of patient identification.
[…] protocol for children with URTI (upper respiratory tract infection).	Yes	Important information to avoid surgical cancellations on the day of the procedure.
[…] it is mentioned that the child may become tearful and irritable; it would be interesting to explain why the child might act this way. It is common for mothers to be startled by this.	Yes	The reasons for postoperative agitation were briefly explained.
[…] the Anesthetic Consent Form is missing.	Yes	Added due to the relevance of the document.
[…] another part where the video could be modified is the illustration of the anesthesia machine/monitor (it is represented as a computer).	Yes	The animation was changed to be more realistic.

### Step 5: target audience validation

For target audience validation, 15 caregivers of hospitalized pediatric patients were invited. Twelve were included, while three were excluded for not meeting the eligibility criteria. Participants’ sociodemographic characteristics and the children’s previous surgical and anesthetic history are presented in Supplementary Table S6.

Cronbach’s alpha was 0.750. Among caregivers, I-CVI values ranged from 91.7% to 100%, with mean values of 97.2% for objectives, 97.2% for organization, 98.6% for style, 97.9% for appearance, and 98.6% for motivation. Domain-level 95% CIs ranged from 85.5–99.9% to 92.5–100.0%. The overall S-CVI/Ave was 98.0% (95% CI: 95.7–99.3%), and the S-CVI/UA was 76.0%. Modified kappa values ranged from 0.916 to 1.000. Items with 11 favorable ratings had an I-CVI of 91.7% and a κ* of 0.916, whereas items with universal agreement had values of 100%. Despite the wide intervals due to the small sample size, the estimates were high across all domains (Table 3). The suggested modifications are presented in Table 2.

**Table 3:** Content validity indices and modified kappa coefficients for the target audience.

Item	CVI (%)	95% CI (%)	Modified kappa, κ*
**VIDEO OBJECTIVES**	**97.2**	**85.5–99.9**	**—**
1.1) Does the video meet the families' objectives?	100.0	73.5–100.0	1.000
1.2) Can the video help you better understand preoperative care and anesthesia?	100.0	73.5–100.0	1.000
1.3) Is the video suitable for use by any professional working with families of children in the preoperative period?	91.7	61.5–99.8	0.916
**ORGANIZATION**	**97.2**	**90.3–99.7**	**—**
2.1) Is the video appealing and does it clearly indicate the material's content?	100.0	73.5–100.0	1.000
2.2) Are the title and content sizes in the scenes adequate?	100.0	73.5–100.0	1.000
2.3) Does the video follow a logical sequence?	100.0	73.5–100.0	1.000
2.4) Is there a clear connection between the information presented in the video?	91.7	61.5–99.8	0.916
2.5) Is the video duration adequate?	91.7	61.5–99.8	0.916
2.6) Do the themes addressed represent important aspects?	100.0	73.5–100.0	1.000
**VIDEO STYLE**	**98.6**	**92.5–100.0**	**—**
3.1) Is the writing style appropriate?	100.0	73.5–100.0	1.000
3.2) Is the text interesting? Is the video's tone friendly?	100.0	73.5–100.0	1.000
3.3) Is the vocabulary accessible to everyone?	100.0	73.5–100.0	1.000
3.4) Is each scene's theme properly associated with its corresponding text?	91.7	61.5–99.8	0.916
3.5) Is the video text clear?	100.0	73.5–100.0	1.000
3.6) Does the video's discourse style correspond to the family members' level of knowledge?	100.0	73.5–100.0	1.000
**APPEARANCE**	**97.9**	**88.9–99.9**	**—**
4.1) Do the video scenes appear organized?	100.0	73.5–100.0	1.000
4.2) Are the illustrations simple?	100.0	73.5–100.0	1.000
4.3) Do the images complement the text?	91.7	61.5–99.8	0.916
4.4) Are the images expressive and sufficient?	100.0	73.5–100.0	1.000
**MOTIVATION**	**98.6**	**92.5–100.0**	**—**
5.1) Is the video appropriate for you?	100.0	73.5–100.0	1.000
5.2) Is the video content presented in a logical manner?	100.0	73.5–100.0	1.000
5.3) Does the text invite interaction? Does it suggest actions?	100.0	73.5–100.0	1.000
5.4) Does the video address the necessary topics for the preoperative preparation of children and their families?	100.0	73.5–100.0	1.000
5.5) Does it invite/prompt changes in behavior and attitude?	100.0	73.5–100.0	1.000
5.6) Does the video provide you with new knowledge?	91.7	61.5–99.8	0.916
**S-CVI/Ave**	**98.0**	**95.7–99.3**	**—**
**S-CVI/UA**	**76.0**	**—**	**—**

Note: I-CVI, Item-level Content Validity Index; S-CVI/Ave, Scale-level Content Validity Index using the average method; S-CVI/UA, Scale-level Content Validity Index using the universal agreement method; CI, confidence interval; κ*, modified kappa coefficient. I-CVI was based on ratings of Totally Adequate or Adequate, and domain values represent mean I-CVIs. Exact binomial 95% CIs were calculated for individual items and pooled favorable ratings for domain and scale estimates. κ* was calculated only for individual items; —, not applicable. Complete response distributions are provided in Supplementary Table S8. N = 12 caregivers.

### Step 6: video finalization

**Figure 1:**
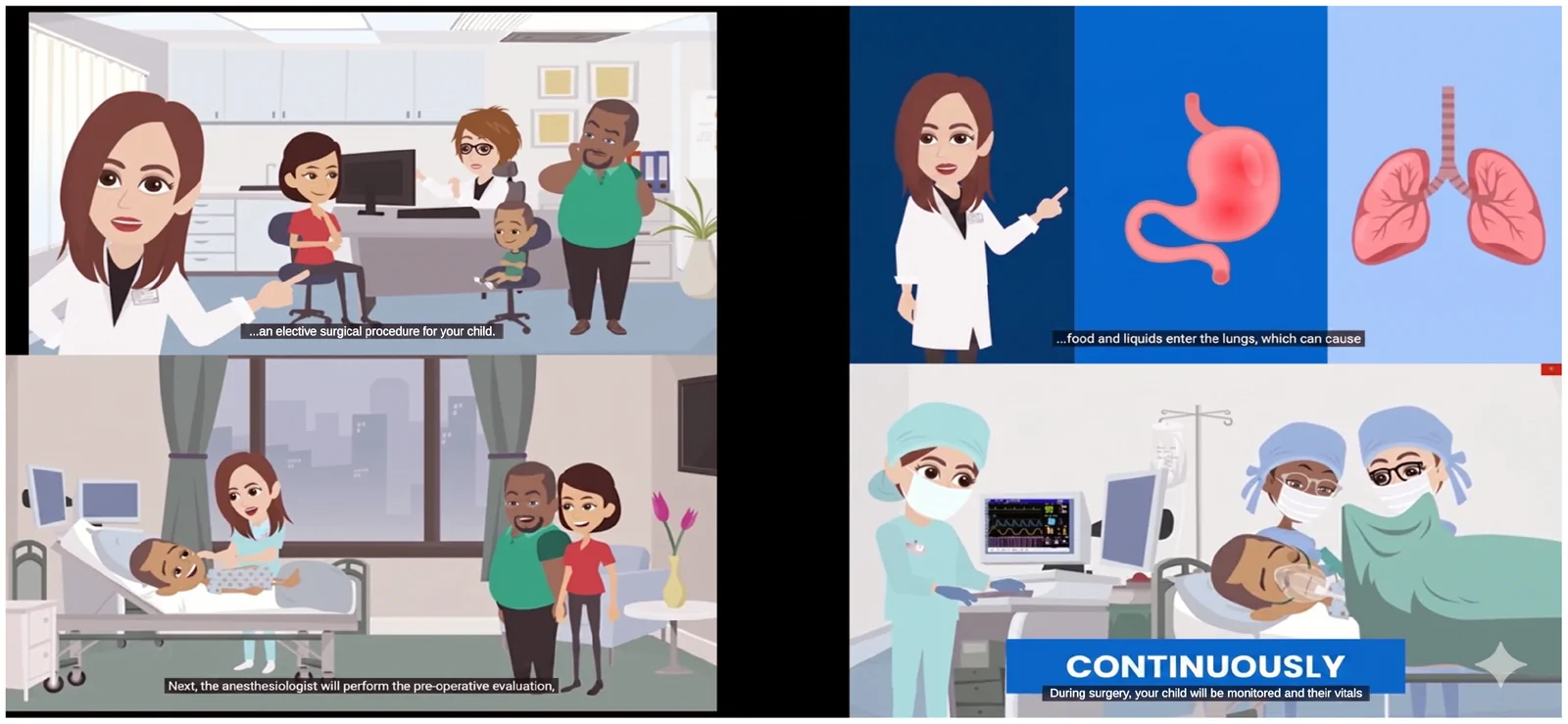
Images from the video “Guidance for Caregivers on Pediatric Anesthesia,” with English translation for publication.

After validation by the expert panel and the target audience, the final version of the video was finalized in 4 min and 40 s (Figure 1). Recommendations from both groups were analyzed and incorporated when relevant, while preserving the original content objectives.

## Discussion

Pediatric perioperative communication remains challenging, particularly regarding fasting, types of anesthesia, monitoring, and postoperative adverse events, which have also been reported as common concerns in other settings [19]. Educational videos may improve comprehension and reduce anxiety among caregivers and children [24,25], which has been associated in the literature with lower analgesic use, less agitation or delirium, and shorter hospital stays [26,27]. However, these outcomes were not evaluated. This study demonstrated content validity, semantic and face adequacy, and acceptability but did not establish clinical effectiveness.

International pediatric anesthesia videos have been reported [5,6,28]. However, none of the identified studies described a formal validation process involving both experts and caregivers. Therefore, direct comparisons of CVI, modified kappa, and Cronbach’s alpha were not possible.

Validation followed the framework proposed by Teixeira and Mota [14], with experts selected using adapted Fehring criteria [13,29]. Experts and caregivers completed different instruments appropriate to the objectives of each step. The expert instrument assessed objectives, structure and presentation, and relevance, supporting content validity. The caregiver instrument assessed objectives, organization, style, appearance, and motivation, supporting semantic and face adequacy. Therefore, the results are complementary but not directly comparable.

All experts assessed the same version in a single round. The Delphi method was not used because progressive consensus was not sought. The indices generally exceeded the adopted cutoff points, and relevant suggestions were incorporated. However, voluntary participation and nonresponse among invited experts may have introduced selection bias.

The four-point Likert scale, with no neutral option, encouraged clear judgments and facilitated dichotomization for the CVI but may have limited the expression of uncertainty and contributed to high indices [15]. The results were consistent with the validation practices mapped by Santos et al. [30], whose scoping review found that CVI predominated, whereas alpha and kappa were less frequently reported. The S-CVI/Ave was 94% among experts and 98% among caregivers, supporting content validity and semantic and face adequacy, respectively. The lower S-CVI/UA among experts does not contradict these findings because this measure requires unanimous agreement and becomes more conservative as the number of evaluators increases [15]. The modified kappa coefficient confirmed agreement beyond chance [16].

Sociocultural appropriateness was the only item below the cutoff point, prompting the simplification of terminology and adjustments to the language and presentation. The 95% CIs increased transparency, although some were wide, particularly among caregivers, reflecting the small sample size. Cronbach’s alpha indicated high internal consistency among experts and acceptable internal consistency among caregivers. Values above 0.90 may suggest redundancy, but the instrument was not shortened because all domains were considered relevant. Among caregivers, 12 participants responded to 25 items, yielding a participant-to-item ratio of 0.48:1, which may make the alpha value of 0.75 unstable. This analysis was complementary because this step was not intended to provide robust psychometric validation. Moreover, understandability and actionability were not assessed using a structured instrument.

The video provides standardized, clear, and accessible information and may serve as the first step in a tiered communication strategy, helping prepare families, facilitate communication, and reduce the need for repetitive guidance [3,6]. However, it does not replace individualized preanesthetic assessment, which is necessary to discuss risks, clinical conditions, and specific concerns [19].

Validation at a single hospital, production in Brazilian Portuguese, and convenience sampling may limit generalizability and applicability. Use in other settings requires linguistic, cultural, educational, and institutional adaptation and revalidation. Dependence on internet access and electronic devices may also restrict accessibility.

Caregivers evaluated the video during hospitalization, although it is intended for use before hospital admission or during the preanesthetic consultation. Perioperative experience may have helped participants assess its usefulness but may also have influenced their responses, limiting external validity.

The exclusion of individuals who were illiterate or had cognitive impairment or communication difficulties reduced the representativeness of groups that could potentially benefit from audiovisual resources. Literacy, cognition, and communication were assessed pragmatically without validated instruments. Moreover, educational attainment alone does not determine beliefs about anesthesia, which are also influenced by personal experiences, culture, family, media, and communication with healthcare professionals [2]. Future studies should include participants with limited literacy and communication needs using adapted or assisted assessment strategies.

The qualitative step included a small number of participants from a single institution, although saturation was monitored and confirmed [9]. The frequencies represent recurrence within the sample rather than population prevalence [12]. Limited reflexivity may have influenced data collection and interpretation. Coding by a single researcher prevented the assessment of intercoder reliability, although the categories and interpretations were discussed with a senior researcher.

Future studies should evaluate the video before hospital admission in larger and more diverse samples, test adaptations in other institutions, and investigate comprehension, anxiety, satisfaction, communication, and family involvement. They should also examine its effects on analgesic requirements, agitation or delirium, and length of hospital stay.

The evaluated version of the educational video presents clear, relevant, and appropriate content to guide caregivers of pediatric patients regarding anesthesia and may serve as a complementary communication tool in the perioperative setting. Its adaptation and local evaluation are recommended before use in other settings, as are studies investigating its clinical effectiveness.

## Conflicts of interest

The authors declare no conflicts of interest.

## Appendix Supplementary materials

Image, application 1

## Footnotes

Supplementary material associated with this article can be found in the online version at doi:10.1016/j.jped.2026.101602.
