# Supplementary material for: Humanizing pediatric anesthesia care: development and validation of an educational video for caregivers
Source: J Pediatr (Rio J). 2026 Sep 8;102(6):101602. doi: 10.1016/j.jped.2026.101602 (PMC13578381; doi:10.1016/j.jped.2026.101602)
Supplement: Supplementary file 1 [file mmc1.docx]

**JPED-D-26-00215_Supplementary Material**

**Table S1. Guideline for Reporting Evidence-Based Practice Educational Interventions and Teaching (GREET) checklist for reporting the educational intervention.**

| **GREET item** | **Description according to GREET** | **Information reported in the present study** | **Manuscript page/location** |
| --- | --- | --- | --- |
| **1. Intervention** | Briefly describe the educational intervention for all groups involved. | Educational video on pediatric anesthesia for caregivers, developed to guide families regarding the perioperative anesthetic process. | Abstract, p. 1; Introduction, p. 2; Methods, pp. 2–3; Results, pp. 3–4 |
| **2. Theory** | Describe the educational theory, concept, or approach used in the intervention. | No formal educational theory was adopted. The intervention was guided by caregivers’ informational needs, principles of health education, accessible communication, and the use of audiovisual resources to facilitate understanding. | Methods, pp. 2–3; Results, pp. 3–4; Discussion, pp. 4–5 |
| **3. Learning objectives** | Describe the learning objectives for all groups involved in the educational intervention. | To provide clear and accessible information to caregivers about pediatric anesthesia, including fasting, type of anesthesia, intraoperative monitoring, emergence from anesthesia, pain, post-anesthesia recovery, and frequent perioperative doubts. | Abstract, p. 1; Introduction, p. 2; Methods, p. 2; Results, pp. 3–4 |
| **4. EBP content** | List the foundation steps of evidence-based practice included in the educational intervention. | Partially applicable. The video was not designed to teach evidence-based practice to health professionals. However, its content was developed based on semistructured interviews with caregivers, literature review, scientific recommendations on pediatric anesthesia, and institutional clinical experience. | Methods, pp. 2–3; Results, pp. 3–4 |
| **5. Materials** | Describe the specific educational materials used in the intervention, including materials provided to learners and those used to train intervention providers. | The educational materials included a script, storyboard, 2D animation, illustrations, inserted texts, narration/voice-over, subtitles, and the final video entitled “Guidance for Caregivers on Pediatric Anesthesia.” | Methods, p. 3; Results, p. 4; Table 2, p. 6; Figure 1, p. 8 |
| **6. Educational strategies** | Describe the teaching and learning strategies used in the educational intervention. | Audiovisual educational strategy using 2D animation, accessible language, narration, subtitles, short written texts, and visual representation of the child’s perioperative anesthetic pathway. | Methods, p. 3; Results, p. 4; Discussion, pp. 4–5; Table 2, p. 6 |
| **7. Incentives** | Describe any incentives or reimbursements provided to learners. | No financial incentives or reimbursements were provided to participants | Not applicable |
| **8. Instructors** | Describe the professional discipline, teaching experience, expertise, and specific training of each instructor involved in the intervention. | The intervention was video-based and did not depend on an instructor for delivery. The content was developed by the research team, and the video was produced by a professional audiovisual company. | Methods, pp. 2–3; Results, p. 4 |
| **9. Delivery** | Describe the mode of delivery of the educational intervention, including whether it was delivered individually or in groups and the learner-to-instructor ratio. | The intervention was delivered as an educational video. During target-audience validation, the video was shown individually to caregivers using an electronic device. There was no instructor during delivery. | Methods, p. 3; Results, p. 4 |
| **10. Environment** | Describe the relevant physical learning spaces where the teaching and learning occurred. | The study was conducted in a Brazilian tertiary pediatric university hospital, with validation performed among caregivers of pediatric patients admitted for surgical treatment. | Methods, pp. 2–3; Results, p. 4 |
| **11. Schedule** | Describe the intervention schedule, including number of sessions, frequency, timing, and duration. | The intervention consisted of a single exposure to the educational video. The preliminary version lasted 3 minutes and 40 seconds; after modifications, the final version lasted 4 minutes and 40 seconds. | Abstract, p. 1; Methods, p. 3; Results, p. 4 |
| **12. Learner time** | Describe the amount of time learners spent in face-to-face contact with instructors and/or in self-directed learning activities. | There was no face-to-face contact with an instructor. Learner time corresponded to individual exposure to the video during target-audience validation. | Methods, p. 3; Results, p. 4 |
| **13. Planned changes/adaptations** | State whether the educational intervention required specific adaptation for learners and describe the adaptations made. | The video was planned for caregivers of children undergoing elective surgical procedures. Adaptations included accessible language, illustrations, subtitles, simple narration, and content defined according to the doubts and informational needs of the target audience. | Methods, pp. 2–3; Results, pp. 3–4; Table 2, p. 6 |
| **14. Unplanned modifications** | State whether the educational intervention was modified during the study and describe what changed, why, when, and how. | Modifications made during development and validation included adjustments to language, illustrations, subtitles, clothing, toys, explanations about anesthesia, and other visual elements. | Results, p. 4; Table 2, p. 6 |
| **15. Attendance** | Describe learner attendance or exposure, how it was assessed, and any strategies used to facilitate attendance. | Twelve caregivers participated in target-audience validation and individually watched the video before completing the evaluation instrument. | Methods, p. 3; Results, p. 4 |
| **16. Planned delivery** | Describe any processes used to determine whether the materials and educational strategies were delivered as originally planned. | During validation, the video was presented as planned. The adequacy of the educational materials and strategies was assessed by expert judges and the target audience using structured instruments, the Content Validity Index, Cronbach’s alpha, and qualitative suggestions. | Methods, p. 3; Results, pp. 4–7 |
| **17. Actual delivery** | Describe the extent to which the number of sessions, frequency, timing, and duration of the educational intervention were delivered as scheduled. | The intervention was delivered as a single exposure to the video during target-audience validation. Routine clinical implementation was not evaluated, as the study focused on the development and validation of the educational material. | Methods, p. 3; Results, p. 4; Discussion (limitations), pp. 7 and 9 |

**Note:** GREET, Guideline for Reporting Evidence-Based Practice Educational Interventions and Teaching; EBP, evidence-based practice. Items related to instructors, repeated sessions, incentives, or formal EBP teaching were considered not applicable or were adapted to the video-based intervention. Information not included in the main manuscript is reported in this supplementary table.

**Table S2. Consolidated Criteria for Reporting Qualitative Research (COREQ) checklist.**

| **No.** | **COREQ item** | **Information reported in the study** | **Reported on page/location** |
| --- | --- | --- | --- |
| **Domain 1: Research team and reflexivity** | | | |
| 1 | Interviewer/facilitator | The semistructured interviews were conducted by the principal researcher. | Manuscript, p. 2; Supplementary Table S2 |
| 2 | Credentials | The principal researcher was a master’s student in the Graduate Program in Surgical Sciences at the Federal University of Rio de Janeiro. | Supplementary Table S2 |
| 3 | Occupation | At the time of the study, the principal researcher was an anesthesiologist at the institution where the study was conducted and was also responsible for developing and validating the educational video as part of her master’s research. | Manuscript, p. 2; Supplementary Table S2 |
| 4 | Gender | The principal researcher, who conducted the interviews, was female. | Supplementary Table S2 |
| 5 | Experience and training | The principal researcher had prior clinical experience as an anesthesiologist but had no formal training in qualitative interviewing. Before data collection and analysis, she undertook self-directed study on qualitative research methods and was guided by the research team regarding the conduct of semistructured interviews and thematic content analysis. Data analysis followed Bardin’s methodological framework and was supported by ATLAS.ti® software. | Manuscript, p. 2; Supplementary Table S2 |
| 6 | Relationship established | The researcher approached the caregivers during the preoperative period. The researcher did not participate in the direct care of the patients included in the study. | Manuscript, p. 2; Supplementary Table S2 |
| 7 | Participant knowledge of the interviewer | Participants were informed about the study objectives before agreeing to participate and signing the informed consent form. | Manuscript, p. 2; Supplementary Table S2 |
| 8 | Interviewer characteristics | The principal researcher was an anesthesiologist familiar with the clinical setting but was not involved in the direct care of the participants. This professional background may have influenced the conduct of the interviews and interpretation of the findings; limited reflexivity was acknowledged as a study limitation. | Manuscript, pp. 2 and 9; Supplementary Table S2 |
| **Domain 2: Study design** | | | |
| 9 | Methodological orientation and theory | The qualitative phase was exploratory and descriptive, using semistructured interviews. Data analysis was performed according to Bardin’s thematic content analysis. | Manuscript, p. 2; Supplementary Table S2 |
| 10 | Sampling | Caregivers were recruited by convenience sampling among those who met the eligibility criteria, and recruitment continued until thematic saturation was reached. | Manuscript, p. 2; Supplementary Table S2 |
| 11 | Method of approach | Caregivers were approached in the Inpatient Unit while waiting for their child's surgical procedure. After clarifying the study objectives and resolution of doubts, they received and signed the informed consent form. | Manuscript, p. 2; Supplementary Table S2 |
| 12 | Sample size | Ten caregivers of pediatric surgical patients participated in the qualitative phase. | Abstract, p. 1; Manuscript, pp. 2–3 |
| 13 | Non-participation | There were no refusals, losses, or withdrawals among the participants invited to take part in the qualitative phase. | Supplementary Table S2 |
| 14 | Setting of data collection | Interviews were conducted in the Inpatient Unit during the preoperative waiting period. | Manuscript, p. 2; Supplementary Table S2 |
| 15 | Presence of non-participants | Only the caregiver and the interviewer were present during the interviews. There was no second interviewer or other non-participants present. | Supplementary Table S2 |
| 16 | Description of the sample | The sample was described according to caregiver age, patient age, relationship to the child, educational level and surgical procedure. | Manuscript, p. 3 |
| 17 | Interview guide | An instrument with closed questions for sociodemographic characterization and open-ended questions about doubts related to the pre-, intra-, and post-anesthetic periods was used. The interview guide was included as Supplementary Appendix 1. | Manuscript, p. 2; Supplementary Appendix 1 |
| 18 | Repeat interviews | No repeat interviews were conducted. Each caregiver was interviewed once. | Supplementary Table S2 |
| 19 | Audio/visual recording | Interviews were audio-recorded after prior authorization from the participants. | Manuscript, p. 2 |
| 20 | Field notes | Field notes were taken during the interviews on the printed interview form, including partial written records of participants’ responses and relevant observations. | Manuscript, p. 2; Supplementary Table S2 |
| 21 | Duration | The interviews lasted between 10 min 02 s and 20 min 48 s, with a mean duration of approximately 15 min 35 s. | Supplementary Table S2 |
| 22 | Data saturation | Data collection was completed after thematic saturation was reached, defined as the point at which new interviews no longer added relevant information to the categories already identified. | Manuscript, p. 2 |
| 23 | Transcripts returned | The interview transcripts were not returned to participants for checking or correction. | Supplementary Table S2 |
| **Domain 3: Analysis and findings** | | | |
| 24 | Number of data coders | Data coding was performed by one researcher, the principal researcher, and the resulting codes and thematic categories were discussed with the research team. | Manuscript, pp. 2 and 9; Supplementary Table S2 |
| 25 | Description of the coding tree | The interviews were coded, with identification of meaning units and grouping into thematic categories related to caregivers’ doubts and informational needs. | Manuscript, pp. 2–3 |
| 26 | Derivation of themes | Themes were derived from participants’ statements through floating reading, coding, identification of meaning units and grouping into thematic categories. | Manuscript, pp. 2–3; Supplementary Table S2 |
| 27 | Software | ATLAS.ti®, version 25.0.1.32924, was used solely to organize, code, and retrieve the qualitative data. Transcriptions were supported by Reshape®. | Manuscript, p. 2; Supplementary Table S2 |
| 28 | Participant checking | The findings were not returned to participants for validation or comments. | Supplementary Table S2 |
| 29 | Quotations presented | Selected representative quotations from caregivers were included to illustrate the main qualitative findings and were identified by participant codes A1 to A10. | Supplementary Table S3 |
| 30 | Data and findings consistent | The qualitative findings were consistent with the data collected and were organized according to recurrent doubts reported by caregivers, including fasting, type of anesthesia, monitoring, postoperative agitation, pain, allergies, and fear of waking up during surgery. | Manuscript, p. 3 |
| 31 | Clarity of major themes | The main themes were clearly presented as caregivers’ doubts and informational needs regarding the perioperative anesthetic process, including preoperative fasting, type of anesthesia, risks, monitoring, emergence from anesthesia, postoperative pain, agitation, and recovery. | Manuscript, p. 3 |
| 32 | Clarity of minor themes | Less frequent but relevant themes, such as allergies, shivering or cold sensation, and fear of waking up during surgery, were described as secondary doubts identified in the interviews. | Manuscript, p. 3 |

**Note:** COREQ, Consolidated Criteria for Reporting Qualitative Research. Items not reported in the main manuscript are documented in this supplementary table. Adapted from Tong A, Sainsbury P, Craig J. Consolidated criteria for reporting qualitative research (COREQ): a 32-item checklist for interviews and focus groups. International Journal for Quality in Health Care. 2007;19(6):349–357.

**Table S3. Qualitative categories, descriptive frequencies, and representative excerpts from caregivers’ interviews.**

| **Thematic category** | **Frequency, n (%)** | **Participant** | **Representative excerpt** | **How the excerpt informed the educational video** |
| --- | --- | --- | --- | --- |
| **Preoperative fasting** | 16 (28.6%) | A5 | “They only told me to fast from midnight onward… I wanted to know why fasting is necessary.” | Explain the reason for fasting, not only the start time. |
|  |  | A7 | “I wanted to know why the fasting period was so long. It was difficult to hold off breastfeeding during the night.” | Clarify fasting intervals according to breastfeeding, formula, solids, and clear liquids. |
|  |  | A2 | “I still had doubts about medications: whether continuous-use medication could be given or not.” | Include guidance that medication use should be clarified before anesthesia. |
|  |  | A4 | “They did not really explain why she had to fast.” | Use simple language to explain the anesthetic risk related to food and liquids. |
|  |  | A8 | “They say, ‘you cannot drink water, you cannot have this,’ and we just obey.” | Promote understanding of fasting instructions rather than passive compliance. |
| **Intraoperative monitoring** | 9 (16.1%) | A3 | “No one explained how heartbeats or oxygenation would be monitored.” | Describe continuous monitoring of heart rate, oxygen saturation, and breathing. |
|  |  | A8 | “I only know from movies… we see it through the machine, the monitors.” | Show monitoring devices in a realistic but non-frightening way. |
|  |  | A10 | “No, I do not know.” | Reinforce that monitoring occurs throughout anesthesia and surgery. |
| **Type of anesthesia** | 9 (16.1%) | A1 | “I would like to know which anesthesia it will be. I do not know if it is spinal anesthesia, vapor… I really do not understand.” | Explain the most common pediatric anesthetic techniques in accessible language. |
|  |  | A9 | “Many people think it is just a little smoke, but they do not know it is general anesthesia.” | Clarify that inhaled anesthetic through a mask may be part of general anesthesia. |
|  |  | A6 | “When they asked which anesthesia he had received, I did not know.” | Name the anesthetic approach and explain it before the procedure. |
| **Postoperative agitation** | 8 (14.3%) | A2 | “I would really like to know, because when he comes back he screams a lot.” | Prepare caregivers for possible emergence agitation and explain that it is usually temporary. |
|  |  | A7 | “The nurse said that if he woke up agitated it would be normal, but I do not know why.” | Explain why agitation may occur and what caregivers can do safely. |
|  |  | A5 | “That scares me a little, because my mother had general anesthesia and woke up delirious.” | Differentiate expected pediatric emergence agitation from severe complications. |
| **Postoperative shivering/cold sensation** | 5 (8.9%) | A2 | “He also comes back trembling from cold… I wanted to know why.” | Explain shivering after anesthesia and the role of warming measures. |
|  |  | A7 | “I have doubts about the trembling… why does it happen?” | Clarify that shivering may be related to cold sensation and recovery from anesthesia. |
| **Postoperative pain** | 3 (5.4%) | A7 | “I have questions, so I know how to take care of him in that part.” | Include practical guidance on pain control, analgesics, blocks, and caregiver observation. |
| **Allergies** | 3 (5.4%) | A4 | “I do not know what type of anesthesia she will receive… if she has an allergy, it could complicate things.” | Address fears of anesthetic allergy and explain team preparedness in reassuring language. |
| **Intraoperative awakening/awareness** | 3 (5.4%) | A3 | “I have many doubts… what would happen if he woke up during the surgery?” | Address fear of waking up during the procedure and explain continuous anesthetic care. |

**Note:** Frequencies refer to 56 coded units of meaning, not to the number of participants; one participant could contribute more than one coded unit and to more than one category. Percentages were calculated using 56 units and may not total exactly 100% because of rounding. Excerpts were translated from Portuguese into English and lightly edited for readability while preserving participants’ intended meaning. Participant codes are anonymized. n, number of coded units of meaning.

**Table S4. Summary of the studies and sources used to support the educational video content and contextualize international video-based interventions.**

| **Thematic category** | **Author and year** | **Title** | **Study or source type** | **Contribution to the video** |
| --- | --- | --- | --- | --- |
| **Informational needs and educational strategies for family members/caregivers** | Bellew et al., 2002 | The introduction of a paediatric anaesthesia information leaflet: an audit of its impact on parental anxiety and satisfaction | Audit/educational intervention study | Demonstrated the importance of providing parents with clear information about anesthesia, transfer to the operating room, recovery, and pain management, as well as evaluating the impact of an information leaflet on parental anxiety and satisfaction. |
|  | Wisselo et al., 2004 | Providing parents with information before anaesthesia: what do they really want to know? | Observational questionnaire-based study | Identified the information parents wished to receive before anesthesia, including premedication, anesthetic induction, adverse effects, postoperative pain management, and their preferred formats for receiving information. |
|  | Sartori et al., 2015 | ¿Qué información preoperatoria desean los padres de niños que serán operados? | Cross-sectional study | Identified the preoperative information most desired by parents, including anesthesia, fasting, medications, complications, pain, and recovery, directly supporting the selection of topics included in the video. |
|  | Bogusaite et al., 2018 | Evaluation of Preoperative Information Needs in Pediatric Anesthesiology | Cross-sectional study | Identified informational needs before pediatric anesthesia, highlighting recovery from anesthesia, postoperative care, pain, and procedure duration as relevant topics for families. |
|  | Aranha and Dsouza, 2019 | Preoperative information needs of parents: a descriptive survey | Descriptive study | Reinforced parents’ need for preoperative information, particularly regarding postoperative care, surgical and anesthetic procedures, and practical guidance. |
|  | Brondani and Fuganti, 2022 | Estratégias educativas pré-operatórias para pais de crianças submetidas a cirurgia ambulatorial: revisão integrativa | Integrative review | Identified videos, brochures, applications, games, surgical tours, virtual reality, music therapy, and other educational approaches used to prepare parents for pediatric outpatient surgery. The findings supported the choice of video as an educational strategy and indicated potential benefits for reducing parental anxiety and improving knowledge and satisfaction. |
|  | Sjauw et al., 2023 | The eXpectations of Parents regarding Anesthesiology Study (XPAS) from a parental perspective: a two-phase observational cross-sectional cohort study | Two-phase observational cross-sectional cohort study | Demonstrated gaps in parents’ knowledge of pediatric anesthesiology and a greater demand for information about the intended effects and potential side effects of anesthesia. |
| **International pediatric anesthesia video interventions** | McEwen et al., 2007 | The effect of videotaped preoperative information on parental anxiety during anesthesia induction for elective pediatric procedures | Randomized controlled trial | Evaluated videotaped preoperative information for parents and its effect on parental anxiety during anesthetic induction, providing an early example of video-based preparation for elective pediatric procedures. |
|  | Rudravaram et al., 2024 | Evaluation of parental anxiety following three methods of pre-anesthesia counseling: Video, brochure and verbal communication | Comparative prospective study | Compared video-based, brochure-based, and verbal pre-anesthesia counseling and demonstrated that video and brochure interventions were associated with greater reductions in parental anxiety than verbal communication alone. These findings contextualized the use of an educational video as a complementary preoperative counseling strategy. |
|  | Brown et al., 2025 | The effect of a caregiver preparation video (Take5) on child anxiety during anesthetic induction: a randomized controlled trial | Randomized controlled trial | Evaluated a caregiver preparation video and its effect on child anxiety during anesthetic induction, contextualizing the use of caregiver-directed audiovisual preparation in pediatric anesthesia. |
| **Technical and scientific foundation in pediatric anesthesia** | Coté, Lerman, and Anderson, 2025 | A Practice of Anesthesia for Infants and Children | Specialized textbook | Served as a reference source for preanesthetic assessment, general and inhalational anesthesia, perioperative analgesia, regional anesthesia, anesthetic safety, and post-anesthesia recovery. |
|  | Frykholm et al., 2022 | Pre-operative fasting in children: guideline from the European Society of Anaesthesiology and Intensive Care | Clinical guideline | Supported the guidance on preoperative fasting, including recommendations concerning clear fluids, breast milk, formula, solid foods, and anesthetic safety. |
|  | Gan et al., 2020 | Fourth Consensus Guidelines for the Management of Postoperative Nausea and Vomiting | International consensus guideline | Provided the basis for information on postoperative nausea and vomiting, including prevention, risk factors, and treatment in the perioperative setting. |
|  | Stepanovic et al., 2024 | Review of the management of children with upper respiratory tract infections in the anesthetic setting | Clinical review/update | Supported the recommendation that caregivers report respiratory symptoms, fever, cough, runny nose, or wheezing before surgery because of the associated risk of perioperative respiratory adverse events. |
|  | Mason, 2017 | Review of pediatric emergence delirium | Narrative review | Supported the explanation provided to caregivers regarding crying, agitation, or confusion during emergence from anesthesia, which are generally transient. |

**Note:** The table summarizes studies and technical sources used in the narrative literature review to support development of the educational video and contextualize international video-based interventions in pediatric anesthesia; it is not intended as an exhaustive or systematic evidence synthesis. **Source:** Prepared by the authors (2025).

**Table S5. Summary characteristics of the expert panel (N = 22).**

| **Variable** | **Category** | **n** | **%** |
| --- | --- | --- | --- |
| **Age group** | 20 to 30 years | 3 | 13.6% |
|  | 31 to 40 years | 6 | 27.3% |
|  | 41 to 50 years | 8 | 36.4% |
|  | Over 51 years | 5 | 22.7% |
| **Gender** | Female | 14 | 63.6% |
|  | Male | 8 | 36.4% |
| **Academic degree** | PhD | 8 | 36.4% |
|  | Specialist | 7 | 31.8% |
|  | Master’s degree | 5 | 22.7% |
|  | Postdoctoral researcher | 2 | 9.1% |
| **Reported expertise profile** | Anesthesiologist | 11 | 50.0% |
|  | Health education and educational technology faculty/researchers | 4 | 18.2% |
|  | Healthcare professional specialized in pediatric surgical care | 3 | 13.6% |
|  | Healthcare professional specialized in child health | 3 | 13.6% |
|  | Healthcare professional specialized in patient safety | 1 | 4.5% |
| **Area of contribution to validation** | Anesthesia / perioperative care | 11 | 50.0% |
|  | Pediatric surgery / perioperative care | 3 | 13.6% |
|  | Health education / educational technology / research | 4 | 18.2% |
|  | Child health / pediatrics | 3 | 13.6% |
|  | Patient safety | 1 | 4.5% |
| **Professional experience** | 5 years | 4 | 18.2% |
|  | 6 to 10 years | 5 | 22.7% |
|  | 11 to 15 years | 3 | 13.6% |
|  | 16 to 20 years | 1 | 4.5% |
|  | More than 20 years | 9 | 40.9% |
| **Adapted Fehring score** | Above 5 points | 15 | 68.2% |
|  | ≤5 points; included based on clinical experience | 7 | 31.8% |
| **Fehring score distribution** | 4 | 2 | 9.1% |
|  | 5 | 5 | 22.7% |
|  | 6 | 4 | 18.2% |
|  | 7 | 1 | 4.5% |
|  | 8 | 2 | 9.1% |
|  | 9 | 8 | 36.4% |

**Note:** Data are presented as n (%) unless otherwise indicated. Percentages were calculated using N = 22 and may not total exactly 100% because of rounding. Seven experts with an adapted Fehring score ≤5 were included based on relevant clinical experience.

**Table S6. Sociodemographic characteristics of participating caregivers and previous information on the pediatric patients. Rio de Janeiro, Brazil, 2025.**

| **Variable** | **Category** | **n** | **%** |
| --- | --- | --- | --- |
| **Caregiver age** | Median, years (min–max) | 34 (20–57) | — |
|  | 20–29 years | 2 | 16.7 |
|  | 30–39 years | 8 | 66.7 |
|  | ≥40 years | 2 | 16.7 |
| **Gender** | Female | 11 | 91.7 |
|  | Male | 1 | 8.3 |
| **Educational level** | Completed elementary school | 2 | 16.7 |
|  | Incomplete high school | 1 | 8.3 |
|  | Completed high school | 6 | 50.0 |
|  | Technical education | 1 | 8.3 |
|  | Completed higher education | 2 | 16.7 |
| **Caregiver was the child’s primary caregiver** | Yes | 11 | 91.7 |
|  | No | 1 | 8.3 |
| **Caregiver’s relationship with the child** | Mother | 10 | 83.3 |
|  | Father | 1 | 8.3 |
|  | Godmother | 1 | 8.3 |
| **Reported municipality/place of residence** | Rio de Janeiro | 9 | 75.0 |
|  | São Gonçalo | 1 | 8.3 |
|  | Nova Iguaçu | 1 | 8.3 |
|  | Duque de Caxias/Xerém | 1 | 8.3 |
| **Child’s previous surgical/anesthetic history** | Previous surgery reported | 4 | 33.3 |
|  | Previous procedure or sedation, with no surgery specified | 2 | 16.7 |
|  | First surgery | 1 | 8.3 |
|  | Not reported | 5 | 41.7 |

**Note:** Data are presented as n (%) unless otherwise indicated. Percentages were calculated using N = 12. The dash indicates not applicable. Previous surgical or anesthetic history was classified according to caregiver reports; “not reported” indicates that no information was available.

**Table S7. Content validity indices and modified kappa coefficients for the expert panel.**

| **Item** | **Totally Adequate** | **Adequate** | **Partially Adequate** | **Inadequate** | **CVI (%)** | **95% CI (%)** | **Modified kappa, κ*** |
| --- | --- | --- | --- | --- | --- | --- | --- |
| **OBJECTIVES** | | | | | **97.3** | **92.2–99.4** | **—** |
| 1.1) The information/content of the video is consistent with the daily needs of families in the preoperative period. | 16 | 5 | 1 | 0 | 95.5 | 77.2–99.9 | 0.955 |
| 1.2) The information/content is important for the relatives of patients in the preoperative period. | 19 | 3 | 0 | 0 | 100.0 | 84.6–100.0 | 1.000 |
| 1.3) The video encourages and/or prompts changes in behavior and attitude among families in the preoperative period. | 16 | 6 | 0 | 0 | 100.0 | 84.6–100.0 | 1.000 |
| 1.4) The video is suitable for circulation in the scientific community as a health education strategy. | 17 | 4 | 1 | 0 | 95.5 | 77.2–99.9 | 0.955 |
| 1.5) The video meets the objectives of institutions that work with patient families in the preoperative period. | 17 | 4 | 1 | 0 | 95.5 | 77.2–99.9 | 0.955 |
| **STRUCTURE AND PRESENTATION** | | | | | **92.1** | **88.0–95.2** | **—** |
| 2.1) The video is appropriate for families of children undergoing elective surgery in the preoperative period. | 16 | 6 | 0 | 0 | 100.0 | 84.6–100.0 | 1.000 |
| 2.2) The messages are presented in a clear and objective manner. | 16 | 3 | 3 | 0 | 86.4 | 65.1–97.1 | 0.864 |
| 2.3) The information presented in the video is scientifically correct. | 18 | 3 | 1 | 0 | 95.5 | 77.2–99.9 | 0.955 |
| 2.4) The video is appropriate for the sociocultural level of families of children undergoing elective surgery in the preoperative period. | 12 | 5 | 5 | 0 | 77.3 | 54.6–92.2 | 0.771 |
| 2.5) There is a logical sequence of the content proposed in the video. | 18 | 4 | 0 | 0 | 100.0 | 84.6–100.0 | 1.000 |
| 2.6) The information contained in the video is well-structured in terms of grammar and spelling. | 20 | 1 | 1 | 0 | 95.5 | 77.2–99.9 | 0.955 |
| 2.7) The writing and speaking style correspond to the families' level of knowledge. | 11 | 8 | 3 | 0 | 86.4 | 65.1–97.1 | 0.864 |
| 2.8) The sizes of the title, text, and topics are adequate. | 18 | 3 | 1 | 0 | 95.5 | 77.2–99.9 | 0.955 |
| 2.9) The video illustrations are expressive and sufficient. | 18 | 1 | 3 | 0 | 86.4 | 65.1–97.1 | 0.864 |
| 2.10) The video is appropriate. | 15 | 6 | 1 | 0 | 95.5 | 77.2–99.9 | 0.955 |
| 2.11) The video duration is adequate. | 18 | 3 | 1 | 0 | 95.5 | 77.2–99.9 | 0.955 |
| **RELEVANCE** | | | | | **94.5** | **88.5–98.0** | **—** |
| 3.1) The themes addressed in the video depict key aspects that should be reinforced when guiding families of children in the preoperative period of elective surgeries. | 21 | 0 | 1 | 0 | 95.5 | 77.2–99.9 | 0.955 |
| 3.2) The video allows for the generalization and transfer of learning to different contexts, including different health education settings. | 16 | 6 | 0 | 0 | 100.0 | 84.6–100.0 | 1.000 |
| 3.3) The video promotes knowledge construction. | 14 | 7 | 1 | 0 | 95.5 | 77.2–99.9 | 0.955 |
| 3.4) The video addresses the necessary topics for the understanding of families of children in the preoperative period of elective surgeries. | 20 | 1 | 1 | 0 | 95.5 | 77.2–99.9 | 0.955 |
| 3.5) The video is suitable for use by any professional working with families of children in the preoperative period of elective surgeries. | 15 | 4 | 3 | 0 | 86.4 | 65.1–97.1 | 0.864 |
| **S-CVI/Ave** | | | | | **94** | **91.4–95.9** | **—** |
| **S-CVI/UA** | | | | | **23.8** | **—** | **—** |

Note: I-CVI, Item-level Content Validity Index; S-CVI/Ave, Scale-level Content Validity Index using the average method; S-CVI/UA, Scale-level Content Validity Index using the universal agreement method; CI, confidence interval; κ*, modified kappa coefficient. I-CVI was calculated as the proportion of evaluators rating each item as Totally Adequate or Adequate. Domain values represent the mean I-CVI of the corresponding items. Exact binomial 95% confidence intervals were calculated for individual items and from pooled favorable ratings for domain and scale-level estimates. S-CVI/UA was calculated as the proportion of items with universal agreement. Modified kappa was calculated only for individual items. A dash indicates not applicable. N = 22 experts.

**Table S8. Content validity indices and modified kappa coefficients for the target audience.**

| **Item** | **Totally Adequate** | **Adequate** | **Partially Adequate** | **Inadequate** | **CVI (%)** | **95% CI (%)** | **Modified kappa, κ*** |
| --- | --- | --- | --- | --- | --- | --- | --- |
| **VIDEO OBJECTIVES** | | | | | **97.2** | **85.5–99.9** | **—** |
| 1.1) Does the video meet the families' objectives? | 11 | 1 | 0 | 0 | 100.0 | 73.5–100.0 | 1.000 |
| 1.2) Can the video help you better understand preoperative care and anesthesia? | 11 | 1 | 0 | 0 | 100.0 | 73.5–100.0 | 1.000 |
| 1.3) Is the video suitable for use by any professional working with families of children in the preoperative period? | 11 | 0 | 1 | 0 | 91.7 | 61.5–99.8 | 0.916 |
| **ORGANIZATION** | | | | | **97.2** | **90.3–99.7** | **—** |
| 2.1) Is the video appealing and does it clearly indicate the material's content? | 11 | 1 | 0 | 0 | 100.0 | 73.5–100.0 | 1.000 |
| 2.2) Are the title and content sizes in the scenes adequate? | 11 | 1 | 0 | 0 | 100.0 | 73.5–100.0 | 1.000 |
| 2.3) Does the video follow a logical sequence? | 11 | 1 | 0 | 0 | 100.0 | 73.5–100.0 | 1.000 |
| 2.4) Is there a clear connection between the information presented in the video? | 11 | 0 | 1 | 0 | 91.7 | 61.5–99.8 | 0.916 |
| 2.5) Is the video duration adequate? | 9 | 2 | 1 | 0 | 91.7 | 61.5–99.8 | 0.916 |
| 2.6) Do the themes addressed represent important aspects? | 11 | 1 | 0 | 0 | 100.0 | 73.5–100.0 | 1.000 |
| **VIDEO STYLE** | | | | | **98.6** | **92.5–100.0** | **—** |
| 3.1) Is the writing style appropriate? | 11 | 1 | 0 | 0 | 100.0 | 73.5–100.0 | 1.000 |
| 3.2) Is the text interesting? Is the video's tone friendly? | 11 | 1 | 0 | 0 | 100.0 | 73.5–100.0 | 1.000 |
| 3.3) Is the vocabulary accessible to everyone? | 12 | 0 | 0 | 0 | 100.0 | 73.5–100.0 | 1.000 |
| 3.4) Is each scene's theme properly associated with its corresponding text? | 11 | 0 | 1 | 0 | 91.7 | 61.5–99.8 | 0.916 |
| 3.5) Is the video text clear? | 12 | 0 | 0 | 0 | 100.0 | 73.5–100.0 | 1.000 |
| 3.6) Does the video's discourse style correspond to the family members' level of knowledge? | 11 | 1 | 0 | 0 | 100.0 | 73.5–100.0 | 1.000 |
| **APPEARANCE** | | | | | **97.9** | **88.9–99.9** | **—** |
| 4.1) Do the video scenes appear organized? | 12 | 0 | 0 | 0 | 100.0 | 73.5–100.0 | 1.000 |
| 4.2) Are the illustrations simple? | 12 | 0 | 0 | 0 | 100.0 | 73.5–100.0 | 1.000 |
| 4.3) Do the images complement the text? | 11 | 0 | 1 | 0 | 91.7 | 61.5–99.8 | 0.916 |
| 4.4) Are the images expressive and sufficient? | 12 | 0 | 0 | 0 | 100.0 | 73.5–100.0 | 1.000 |
| **MOTIVATION** | | | | | **98.6** | **92.5–100.0** | **—** |
| 5.1) Is the video appropriate for you? | 12 | 0 | 0 | 0 | 100.0 | 73.5–100.0 | 1.000 |
| 5.2) Is the video content presented in a logical manner? | 12 | 0 | 0 | 0 | 100.0 | 73.5–100.0 | 1.000 |
| 5.3) Does the text invite interaction? Does it suggest actions? | 11 | 1 | 0 | 0 | 100.0 | 73.5–100.0 | 1.000 |
| 5.4) Does the video address the necessary topics for the preoperative preparation of children and their families? | 12 | 0 | 0 | 0 | 100.0 | 73.5–100.0 | 1.000 |
| 5.5) Does it invite/prompt changes in behavior and attitude? | 12 | 0 | 0 | 0 | 100.0 | 73.5–100.0 | 1.000 |
| 5.6) Does the video provide you with new knowledge? | 5 | 6 | 1 | 0 | 91.7 | 61.5–99.8 | 0.916 |
| **S-CVI/Ave** | | | | | **98.0** | **95.7–99.3** | **—** |
| **S-CVI/UA** | | | | | **76.0** | **—** | **—** |

Note: I-CVI, Item-level Content Validity Index; S-CVI/Ave, Scale-level Content Validity Index using the average method; S-CVI/UA, Scale-level Content Validity Index using the universal agreement method; CI, confidence interval; κ*, modified kappa coefficient. I-CVI was calculated as the proportion of evaluators rating each item as Totally Adequate or Adequate. Domain values represent the mean I-CVI of the corresponding items. Exact binomial 95% confidence intervals were calculated for individual items and from pooled favorable ratings for domain and scale-level estimates. S-CVI/UA was calculated as the proportion of items with universal agreement. Modified kappa was calculated only for individual items. A dash indicates not applicable. N = 12 caregivers.

**Appendix 1**

**Semistructured Interview Script with Caregivers**

**Participant profile — caregiver and child**

Fictitious name:

Address: ___________________________________________________________

Phone number: ___________________

Age of the caregiver (in years): ______________________________

Age of the child (in years or months): ________________________

Diagnosis: _________________________________________________________

Scheduled surgical procedure: ______________________________________

Degree of relationship to the child: _______________________________________

Are you the child’s primary caregiver? ( ) Yes ( ) No

Level of education: _________________________________________________

**Questionnaire for family members about doubts regarding pediatric anesthesia**

### 1. Before anesthesia

Main question

- Tell me what you know about the care your child needs before anesthesia.

Probing questions (if necessary)

a. What do you know about preoperative fasting?
b. What is your understanding of the required fasting period?
c. What do you think your child can or cannot eat or drink before anesthesia?
d. What is your understanding of whether water is allowed during the fasting period?
e. Do you have any remaining questions or concerns about fasting?
f. How would you describe the information you received about preoperative fasting? What was clear, and what remained unclear?

### 2. About the anesthetic procedure

Main question

- Tell me what you know or expect about the anesthesia your child will receive.

Probing questions

a. What is your understanding of the type of anesthesia that will be used?
b. What do you know about how your child will be monitored during the procedure?
c. Do you have any concerns about your child waking up during the procedure?
d. What comes to mind when you hear the expression "allergy to anesthesia"?
e. What are your main fears or concerns regarding anesthesia?

### 3. After surgery

Main question

- What do you expect will happen after the surgery, when your child wakes up from anesthesia?

Probing questions

a. What do you know about agitation during emergence from anesthesia?
b. What is your understanding of postoperative shivering?
c. What questions or concerns do you have about postoperative pain?

### 4. Educational video

Main question

- If an educational video about pediatric anesthesia were developed for family members, what information would you like it to include?

Probing questions

a. Are there any topics you consider essential?
b. Is there any information that you feel has not been adequately explained?
